# Professionalism Mini-Evaluation Exercise in Finland: A preliminary investigation introducing the Finnish version of the P-MEX instrument

**Published:** 2015-10

**Authors:** MAX KARUKIVI, OUTI KORTEKANGAS-SAVOLAINEN, ULLA SAXÉN, KIRSI-MARIA HAAPASALO-PESU

**Affiliations:** 1 Satakunta Hospital District, Psychiatric Care Division, Harjavalta, Finland;; 2 Department of Psychiatry, Turku University, Turku, Finland;; 3 Medical Education Research and Development Centre, Turku University, Turku, Finland;

**Keywords:** Assessment, Medical students, Professionalism

## Abstract

**Introduction:**

Teaching medical professionalism is increasingly acknowledged as an important aspect of medical education. The Professionalism Mini-Evaluation Exercise (P-MEX) is an assessment tool for evaluating medical professionalism, but no studies using it as a self-assessment instrument have been reported. This paper reports on a preliminary investigation of the Finnish version of the P-MEX instrument as an assessment and self-assessment measure.

**Methods:**

The sample in the present cross-sectional study comprised all 23 medical students and recent graduates (15 females and 8 males) participating in a summer school of psychiatry program in 2014. The two-month program combines clinical work with multifaceted teaching and intensive tutoring. At the end of the program, the participants’ medical professionalism was assessed by the tutors and other members of the work community as well as the students themselves using the Finnish version of the P-MEX instrument. The P-MEX scores were compared, using the Friedman test.

**Results:**

The mean values and SD for the P-MEX assessments were as follows: tutor assessment 3.26±0.21, work community assessment 3.32±0.26 and self-assessment 3.01±0.07. No significant gender differences were observed. The tutor and work community assessments were significantly correlated (r=0.573, p=0.040), but the self-assessment scores did not correlate with either of the other assessments. Overall, the students evaluated their skills significantly poorer in comparison to the other assessments.

**Conclusion:**

Although the small sample size limits the generalization of these preliminary results, the Finnish version of the P-MEX instrument appears to be a feasible measure of medical professionalism. The instrument can also be used as a self-assessment instrument, but subjective evaluations should be complemented with external assessments or feedback in order to take individual and cultural aspects into account.

## Introduction

Medical professionalism is a multi-dimensional concept that encompasses several aspects regarding attitude, conduct and professional responsibilities. In the past decades, the importance of medical professionalism has been recognized and accentuated by the public statements of several renowned organizations (1, 2). The objective to derive these principles to a more practical form has resulted in advances in the definitions and behaviours characterizing medical professionalism (3, 4). In practice, medical professionalism can be broadly defined as a balance between patient centeredness and medical expertise, while taking available resources and societal expectancies into account. Although the relevant principles are widely acknowledged among clinicians, the implementation and integration of the conventions to daily practice calls for more effort than just declarations (5).

Since medical professionalism obviously plays a key role when future professionals form their individual manners in practicing medicine, it is increasingly identified as a fundamental aspect in teaching medical professionals (6). One method to endorse professional behaviour is to assess it, although the number of psychometrically sound instruments is limited (6, 7). Professionalism Mini-Evaluation Exercise (P-MEX) was developed by Cruess et al. (2006) as an assessment tool for medical professionalism (8). The measure was preceded by the mini-Clinical Examination (mini-CEX), which is used to evaluate the clinical skills of students and residents, but it does not include specific observational behaviours associated to medical professionalism (9). Only few studies have been conducted using the P-MEX instrument, but it has shown good validity and reliability (8, 10, 11).

The core skills associated with medical professionalism are, in many ways, pronounced in psychiatry (12). Psychiatric assessment and therapeutic treatment methods are largely based on patient contact and communication skills. In addition to actual psychological distress also ethical questions and several other aspects of life, such as personal history, somatic illnesses, spirituality and social environment typically emerge in psychiatric practice. Furthermore, a social stigma is still associated with many mental illnesses.

To date, there are no feasible assessment methods for medical professionalism in Finnish language. The P-MEX instrument was chosen for the present study due to its confirmed psychometric properties and although being a versatile instrument, it is easy to use. In previous research, the importance of individual reflection and multisource feedback in teaching professionalism has been emphasized (13). However, to our best knowledge, no experiments with the P-MEX instrument as a self-assessment measure have been previously reported. Therefore, this pilot study was conducted for the purpose of evaluating the Finnish translation of the P-MEX instrument and its feasibility as an assessment and self-assessment tool in a sample of medical students working in psychiatry.

## Methods

The sample (n=23) comprises those medical students who participated in a summer school of psychiatry program in June−July 2014. The two-month program has been developed within the Satakunta Hospital District in Finland and is intended for medical students interested in psychiatry. Participation is voluntary and all those interested in the program can apply with an informal application regardless of the university where they study at. The program combines clinical work in the Psychiatric Care Division units with intensive tutoring, weekly teaching sessions with patient case workshops, and leisure activities. The students are paid for their work, and teaching is included in their working hours. The tutors are recruited from the hospital’s own staff, and they are either specialists or experienced residents. Every tutor has one to five participants to guide. The program has been described in detail elsewhere (14). In the sample, two participants had just completed their degree, one had completed five years, thirteen four years and seven three years of studies. Altogether 65% (n=15) of the participants were female. The average age was 25 years both for females (range 22−43 years) and males (range 23−32 years). The participants were thoroughly informed of the study protocol with the possibility to discuss any emerging questions. All of the participants gave their verbal informed consent.

The participants’ medical professionalism was assessed using the P-MEX instrument (8). It comprises 21 items that represent four skill categories: doctor-patient relationship skills, reflective skills, time management, and interprofessional relationship skills (Table 1). Each item is rated on a four-point scale where 1 = unacceptable, 2 = below expectations, 3 = met expectations, and 4 = exceeded expectations. It is also possible to score an item as “not applicable”. In addition to the English version of the instrument (8), the Japanese version has also shown good psychometric properties (10, 11).

**Table 1:** Mean scores and standard deviations (SD) for the Professionalism Mini-Evaluation Exercise (P-MEX) items.

**Skill category**		**Mean±SD**			
**Doctor-patient relationship skills**	**Item**	**Tutor (n=23)**	**Work community (n=13)**	**Self-assessment (n=23)**	P^*^
1.	Listened actively to patient	3.57±0.51	3.54±0.52	3.00±0.00	0.002
2.	Showed interest in patient as a person	3.22±0.42	3.54±0.52	3.09±0.29	0.012
3.	Recognized and met patient needs	3.22±0.42	3.23±0.44	2.96±0.21	0.069
4.	Extended him/herself to meet patient needs	3.13±0.34	3.15±0.38	3.04±0.21	0.78
5.	Ensured continuity of patient care	3.32±0.48	3.23±0.44	2.91±0.29	0.17
6.	Advocated on behalf of a patient	3.14±0.35	3.08±0.29	3.09±0.29	0.78
11.	Maintained appropriate boundaries	3.13±0.34	3.42±0.52	3.00±0.00	0.015
**Reflective skills**					
7.	Demonstrated awareness of limitations	3.30±0.47	3.23±0.44	3.09±0.29	0.45
8.	Admitted errors/omissions	3.22±0.42	3.18±0.41	3.04±0.21	0.22
9.	Solicited feedback	3.43±0.51	3.18±0.41	2.96±0.21	0.009
10.	Accepted feedback	3.43±0.51	3.31±0.48	3.00±0.00	0.030
12.	Maintained composure in a difficult situation	3.30±0.47	3.38±0.51	3.04±0.37	0.044
**Time management**					
14.	Was on time	3.26±0.45	3.31±0.48	2.87±0.34	0.047
15.	Completed tasks in a reliable fashion	3.43±0.51	3.69±0.48	3.09±0.29	0.004
17.	Was available to colleagues	3.17±0.39	3.38±0.51	3.04±0.21	0.015
**Interprofessional relationship skills**					
11.	Maintained appropriate boundaries	3.13±0.34	3.42±0.52	3.00±0.00	0.015
13.	Maintained appropriate appearance	3.13±0.34	3.38±0.51	3.00±0.00	0.015
16.	Addressed own gaps in knowledge and skills	3.39±0.50	3.23±0.44	3.00±0.43	0.030
18.	Demonstrated respect for colleagues	3.22±0.42	3.38±0.51	3.00±0.00	0.042
19.	Avoided derogatory language	3.22±0.42	3.46±0.52	3.04±0.21	0.050
20.	Maintained patient confidentiality	3.04±0.21	3.31±0.48	3.04±0.21	0.074
21.	Used health resources appropriately	3.18±0.40	3.17±0.39	2.90±0.30	0.018

^*^Friedman test

Two of the authors [MK and OKS] translated the P-MEX instrument from English to Finnish and two professional linguists performed a back-translation into English. The translation was then approved by the developer of the instrument (Richard Cruess). The assessments were done by the tutors at the end of the two-month program, and thus, they included observations of a considerable amount of patient encounters, collaboration with different occupational groups, and varied clinical duties. For multisource feedback, the other occupational groups in the students’ work communities also assessed the students for their part. Finally, the participants themselves completed a self-assessment. In individual feedback sessions, the assessments were openly discussed by the tutor and the participant.

The distributions of the variables were skewed, and accordingly, they were treated as non-parametrical. Mann-Whitney U-test was used for comparisons between males and females. The average scores for the P-MEX instrument were compared for the three groups (tutors, work community, self-assessment) using the Friedman test. The low number of subjects precluded the possibility of a confirmatory factorial analysis. The Cronbach’s coefficient alpha scores were used to assess the internal consistency for the P-MEX instrument and the scores showed acceptable results: 0.70 for tutor, 0.90 for work community and 0.63 for self-assessments. 

## Results

The mean values and SD for the P-MEX assessments were as follows: tutor 3.26±0.21, work community 3.32±0.26 and self-assessments 3.01±0.07. The tutor and work community assessment scores correlated significantly (r=0.573, p=0.040), whereas the self-assessment scores did not correlate significantly with either of the other assessments. Item analysis showed statistically significant differences between the assessments (Table 1).

Subjective ratings were consistently poorer than the external assessments. There were no statistically significant differences between genders in the mean and SD values: tutor assessment 3.29±0.22 for females and 3.20±0.20 for males (p=0.43), work community 3.28±0.23 and 3.37±0.30 (p=1.00), and self-assessments 3.01±0.09 and 3.01±0.02 (p=0.43), respectively. 

## Discussion

The main finding of the present study was that the Finnish version of the P-MEX instrument produces similar results compared with the previous studies with larger samples and thus, appears to be a feasible measure of medical professionalism. Regarding the tutor and work community assessments, the previous studies conducted in Canada(8) and Japan (10, 11) have resulted in similar findings, with average scores ranging from 3.23 to 3.25. Although a longer 24-item version was used in these studies, the developers of the instrument support the use of the 21-item version (8). Compared with these previous studies, the number of subjects was markedly lower in the present study, but the main results were in accordance.

Our second main focus was the evaluation of the P-MEX as a self-assessment instrument, which has not been previously reported. While this pilot-type approach precludes the possibility of reliable comparisons in the lack of previous results, the multisource feedback method results in some provisional findings. Overall, the participants themselves assessed their skills as being lower in comparison to the scores given by the other assessors. Of the different skill categories, time management, in particular, was evaluated as a weakness, although significant differences were found in all categories. From a cultural aspect, Finnish people are typically characterized by humility and pronounced modesty, although not in the same lengths as East Asian cultures (15). Thus, it can be hypothesized, that the students’ consistent tendency to evaluate their professionalism skills to be lower in comparison to the other assessors, was, at least partly, explained by an attempt to avoid overestimating their skills. In any case, both medical students and clinicians are known to struggle in making reliable self-assessments (16). Thus, it is plausible that this finding is not an instrument-related issue, which highlights the importance of complementary assessments.

Clinical supervision has been suggested to be a particularly feasible method for the assessment of professionalism (17). Extensive clinical supervision is a core element of the summer school program and in addition to providing observational material for the assessment; it is plausible that the tutors’ example and informal guidance also had an effect on the students’ professionalism. In the feedback sessions, many of the students implied that self-assessment of these types of skills was new to them, which led them to emphasize the neutral “met expectations” alternative. Some evaluators and students found it a bit difficult to determine the expected skill level. This leads us to emphasize that the expectations should be defined with reference to students with equal experience. Unlike the study by Tsugawa et al. (2011) (11), the assessments were not blinded in our study, but instead they were openly discussed in the feedback sessions. This facilitated a mutual elaboration on the different assessments, which was experienced by the students as fruitful. Considering the nature of professionalism, the importance of individual reflection is acknowledged(5) and open discussion may further promote the formation of professionalism skills.

## Conclusion

The limitations of the present study include the small number of subjects, which precluded the possibility of a more in-depth analysis of the psychometric properties of the instrument and limits the generalization of the results. However, both the good internal consistency and equivalent results compared with previous studies support the feasibility of the instrument.

In the present study, the P-MEX was used as a self-assessment instrument for the first time. It is also a potential instrument in this regard, but the lack of comparable studies limits the evaluation of the results based on self-assessments. In any case, according to our experieces, the self-assessments appear to be useful as an additional tool to endorse professionalism, but should be complemented by external assessments and discussion in order to take individual aspects into account. The present findings should be confirmed in larger samples. 
